# Parametric maps of spatial two-tissue compartment model for prostate dynamic contrast enhanced MRI - comparison with the standard Tofts model in the diagnosis of prostate cancer

**DOI:** 10.21203/rs.3.rs-2539644/v1

**Published:** 2023-02-08

**Authors:** Xueyan ZHOU, Xiaobing Fan, Aritrick Chatterjee, Ambereen Yousuf, Tatjana Antic, Aytekin Oto, Gregory S. Karczmar

**Affiliations:** Harbin University; The University of Chicago; The University of Chicago; The University of Chicago; The University of Chicago; The University of Chicago; The University of Chicago

**Keywords:** Dynamic contrast enhanced MRI, standard Tofts model, spatial two-tissue compartment model, prostate cancer

## Abstract

The spatial two-tissue compartment model (2TCM) was used to analyze prostate dynamic contrast enhanced (DCE) MRI data and compared with the standard Tofts model. A total of 29 patients with biopsy-confirmed prostate cancer were included in this IRB-approved study. MRI data were acquired on a Philips Achieva 3T-TX scanner. After T2-weighted and diffusion-weighted imaging, DCE data using 3D T1-FFE mDIXON sequence were acquired pre- and post-contrast media injection (0.1 mmol/kg Multihance) for 60 dynamic scans with temporal resolution of 8.3 s/image. The 2TCM has one fast (K_1_^trans^ and k^1^_ep_) and one slow (K_2_^trans^ and k^2^_ep_) exchanging compartment, compared with the standard Tofts model parameters (K^trans^ and k_ep_). On average, prostate cancer had significantly higher values (p < 0.007) than normal prostate tissue for all calculated parameters. There was a strong correlation (r = 0.94, p < 0.0001) between K^trans^ and K_1_^trans^ for cancer, but weak correlation (r = 0.28, p < 0.05) between k_ep_ and k^1^ep. Average root-mean-square error (RMSE) in fits from the 2TCM was significantly smaller (p < 0.001) than the RMSE in fits from the Tofts model. Receiver operating characteristic (ROC) analysis showed that fast K_1_^trans^ had the highest area under the curve (AUC) than any other individual parameter. The combined four parameters from the 2TCM had a considerably higher AUC value than the combined two parameters from the Tofts model. The 2TCM may be useful for quantitative analysis of prostate DCE-MRI data and may provide new information in the diagnosis of prostate cancer.

## Introduction

Multi-parametric MRI (mpMRI) plays an important role in detection and grading of prostate cancer (PCa) [1–3]. Although T2-weighted (T2W) imaging and diffusion-weighted imaging (DWI) are the two main components of prostate mpMRI, dynamic contrast enhanced (DCE) MRI assists in the interpretation of T2W imaging and DWI in detection of high-risk PCa, and in surveillance status post prostatectomy, radiotherapy or focal ablation [4–9]. However, compared to T2W imaging and DWI, qualitative analysis of DCE-MRI is the least standardized [10]. There are often overlap enhancement patterns between benign and malignant tissues in prostate peripheral and transition zones. Some benign lesions, such as benign prostatic hyperplasia (BPH) nodules, show strong early enhancement, while some malignant lesions do not show early enhancement or washout [11]. Therefore, there are often false positives in diagnosis of PCa by using DCE-MRI [12].

Prostate DCE-MRI data is also often analyzed quantitatively using pharmacokinetic models, such as the standard Tofts model, to extract the volume transfer rate constant (K^trans^) (exchange between blood plasma and the extravascular extracellular space (EES)) and fractional volume of EES (v_e_) [13,14]. However, the standard Tofts model may not be compatible with the heterogeneous characteristics of the tumor micro-environment that results in an initial rapid uptake of contrast agent followed by a less rapid, but prolonged, uptake of the contrast agent [15,16]. As a result, tumor heterogeneity at the microscopic level could cause poor fits to DCE-MRI data for the standard Tofts model and errors in extracting K^trans^ and v_e_. This would limit the diagnostic accuracy when using the standard Tofts model to analyze DCE-MRI data. Therefore, more complex models should be used to analyze DCE-MRI data, such as the multicompetent models [17], “shutter-speed” model [18], two-compartment exchange model 2CXM [19, 20], and spatial two-tissue compartment model (2TCM) [21].

In contrast to the standard Tofts model with only one tissue compartment, the 2TCM has one slow and one fast exchanging tissue compartment. Previous study demonstrated that MRI contrast agent distributions in heterogeneous tissue could be more adequately accessible with 2TCM, especially at tumor margins [21]. In this study, the 2TCM was used to analyze prostate DCE-MRI data and prove the hypothesis that diagnostic accuracy could be improved for DCE-MRI. The results obtained from 2TCM were compared with those from the standard Tofts model as a DCE model evaluation.

## Methods

### Patients

This retrospective study was approved by the Ethics Committee of the University of Chicago, Institutional Review Boards (IRB), with the approval number IRB13–0756. Informed patient consent was compliant with the Health Insurance Portability and Accountability Act (HIPAA). The patients imaged and followed by subsequent radical prostatectomy were recruited for this study between March 2014 and May 2015. Twenty-nine patients (mean age 57 years, range 40–70 years; and mean PSA 7.4 ng/lm, range 1.8–26.1 ng/lm) with biopsy-confirmed prostate cancer were included in this study. Patients who received prior radiation or hormonal therapy were excluded.

### MRI protocol

The mpMRI data were acquired on Philips Achieva 3T-TX scanner (Philips Healthcare, Netherlands) using a 6-channel cardiac phased array coil placed around the pelvis, combined with an endorectal coil (Medrad, Bayer Healthcare, USA). After T2W imaging and DWI, baseline T1 mapping was performed using a variable flip angle (VFA) sequence (TR/TE = 12/2.3 ms, field of view (FOV) = 385×250 mm, matrix size = 308×220, flip angle = 3°, 5°, 10°, 15°, 20°, 30°, slice thickness = 3.5 mm, number of slices = 24, SENSE factor = 1.67, half scan factor = 0.675). Subsequently, DCE data using 3D T1-FFE mDIXON sequence were acquired pre- and post-contrast media injection 0.1 mmol/kg of Multihance (TR/TE1/TE2 = 4.6/1.7/3.3 ms, FOV = 250×385 mm^2^, matrix size = 200×308, flip angle = 10°, slice thickness = 3.5 mm, number of slices = 24, SENSE factor = 1.67, half scan factor = 0.675) for 60 dynamic scans with temporal resolution of 8.3 s/image.

### Data analysis

DCE-MRI data were analyzed using Matlab (Mathworks, Natick, MA, USA) with an in-house software package. H&E-stained whole mount radical prostatectomy sections were matched with corresponding prostate MR images by an expert radiologist (AO – 19 years’ experience with prostate MRI) and expert pathologist (TA – 16 years’ experience) to guide the selection of regions-of-interests (ROIs) of cancer and normal prostate tissues in the peripheral zone (PZ), transition zone (TZ), central zone (CZ), and anterior fibromuscular stroma (AFMS). A medical physicist (XF – 19 years’ experience with DCE-MRI) manually traced ROIs of blood vessels, used for arterial input function (AIF), on the iliac artery on a slice with cancer on DCE images. On slices with ROIs, pixel contrast agent concentration (C(t)) as function of time (t) was calculated from the non-linear model using the gradient echo signal equation [22] with measured T1 value from VFA sequence.

For each C(t), physiological parameters were extracted from the standard Tofts model [13]:

1 $$C(t)={\text{K}}^{\text{trans}}\int_{0}^{t}C_{p}(\tau )\;\text{exp}\;\left(-(t-\tau ){\text{k}}_{\text{ep}}\right)d\tau ,$$

as well as from the 2TCM [21]:

2 $$C(t)=\int_{0}^{t}C_{p}(\tau )\left[{\text{K}}_{1}^{\text{trans}}\;\text{exp}\;\left(-(t-\tau ){\text{k}}_{\text{ep}}^{1}\right)+{\text{K}}_{2}^{\text{trans}}\;\text{exp}\;\left(-(t-\tau ){\text{k}}_{\text{ep}}^{2}\right)\right]d\tau ,$$

where C_p_(t) = C_b_(t)/(1 - Hct) is the AIF, C_b_(t) is contrast media concentration in blood, Hct is the hematocrit (= 0.42), k_ep_=K^trans^/v_e_ and ${\text{k}}_{\text{ep}}^{\text{i}}={\text{K}}_{\text{i}}^{\text{trans}}/{\text{v}}_{\text{e}}^{\text{i}}(\text{i}=1,2)$ is the efflux rate constant from the EES to the plasma. The root-mean-square error (RMSE) was calculated to evaluate data fitting for Tofts and 2TCM models.

In order to obtain unique results for fitting C(t) using MATLAB, Eq. 2 was written in an asymmetric form as follows:

3 $$C(t)={\text{K}}_{1}^{\text{trans}}\int_{0}^{t}C_{p}(\tau )\;\text{exp}\;\left(-(t-\tau ){\text{k}}_{\text{ep}}^{1}\right)•[1+\epsilon •\;\text{exp}\;(-(t-\tau )•\lambda )]d\tau ,$$

where ${\text{K}}_{2}^{\text{trans}}=\varepsilon •{\text{K}}_{1}^{\text{trans}}$ and ${\text{k}}_{\text{ep}}^{2}={\text{k}}_{\text{ep}}^{1}+\lambda$. Please note that for different pixels either ${\text{K}}_{1}^{\text{trans}}$ or ${\text{K}}_{2}^{\text{trans}}$ could be large or small depending on whether ε is smaller or larger than 1.0. In order to consistently compare calculated parameters, we selected ${\text{K}}_{1}^{\text{trans}}$ as the larger one of them $\left(=\text{max}\;\left[{\text{K}}_{1}^{\text{trans}},{\text{K}}_{2}^{\text{trans}}\right]\right)$ with its corresponding ${\text{k}}_{\text{ep}}^{1}$ or ${\text{k}}_{\text{ep}}^{2}$ as ${\text{k}}_{\text{ep}}^{1}$. Therefore, for each pixel ${\text{K}}_{1}^{\text{trans}}$ is always larger than ${\text{K}}_{2}^{\text{trans}}$ in our study.

Parametric maps were only generated for the slices with ROIs. For each ROI, the average value was calculated for all physiological parameters. Generally, PCa shows earlier and faster enhancement and earlier contrast agent washout compared to normal prostate tissue [23]. Unfortunately, high K^trans^ regions were not necessarily indicative of cancer. Based on K^trans^ map and the matching histopathology slice, normal prostate tissues region with high K^trans^ value (similar to nearby cancer) was also manually traced with a slightly larger ROI on the map. Then a cutoff value was used to determine final ‘selected false positive’ ROI of K^trans^. The cutoff value was determined from the average K^trans^ value of a nearby cancer ROI minus its standard deviations. With this method, the selected false positive ROI would have an average K^trans^ value close to the average cancer K^trans^ value.

A paired t-test was performed to determine whether there was a significant difference for the RMSE between these models. Pearson’s correlation coefficient was calculated to test whether there are linear correlations between physiological parameters obtained from the standard Tofts model and 2TCM. One-way ANOVA with post-hoc Tukey HSD (Honestly Significant Difference) Test was performed to determine whether there was significant difference for all calculated physiological parameters between cancer, normal tissue and false positive region. Receiver operating characteristic (ROC) analysis was used to evaluate performance in differentiating between cancer and normal tissue. Binary logistic regression was used to assess combinations of parameters from the 2TCM. All the statistical analysis was calculated using SPSS (IBM Corporation, Armonk, NY).

## Results

**Figure 1:**
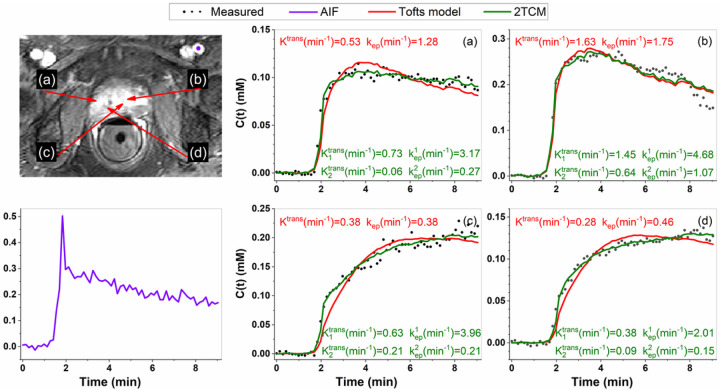
Plot of AIF (purple line) traced over iliac artery (purple dot on image) is shown below DCE image. Plots of measured C(t) (black dots) at selected three pixels in tumor (a, b, and c) and one pixel in normal tissue (d) indicated by red arrows on DCE-MRI, and as well as corresponding fits of C(t) by the Tofts model (red line) and the 2TCM (green line). The extracted parameters are also given within the figure.

A total of 54 index lesion of prostate cancers ROIs (18 Gleason 3 + 3, 28 Gleason 3 + 4, seven Gleason 4 + 3, one Gleason 4 + 5), 83 normal tissue ROIs in different prostate zones (PZ = 22, TZ = 21, CZ = 20, and AFMS = 20), and 24 false positive ROIs were traced. Figure 1 shows a prostate DCE image, plot of the AIF (purple line) and plots of measured C(t) (black dots), as well as corresponding fits with the standard Tofts model (red line) and 2TCM (green line) for three tumor pixels (a, b and c) and one normal tissue pixel (d). It can be seen that the 2TCM fits are much better than those of the standard Tofts model. On average (± standard deviation), the RMSE (= 0.013 ± 0.009) obtained 2TCM was significantly smaller (p < 0.001) than the RMSE (= 0.022 ± 0.024) obtained from the Tofts model, which shows the better fitting for the data using 2TCM.

**Figure 2:**
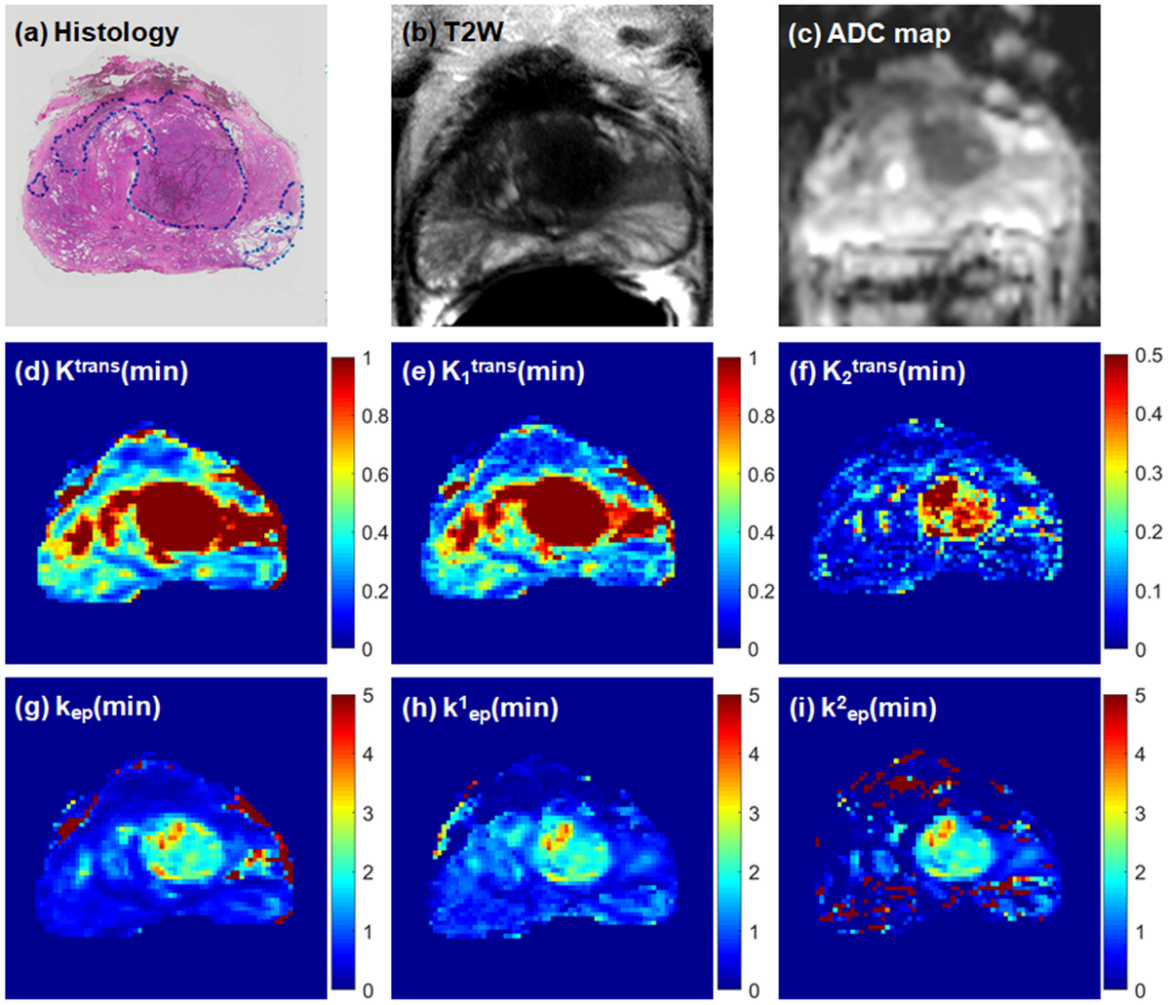
Comparison of parametric maps obtained from the standard Tofts model and 2TCM in a 67-year-old patient with large Gleason 3+4 index lesion: (a) whole-mount histology from prostatectomy specimen with cancer markers, (b) high resolution T2W image, (c) ADC map, (d) K^trans^ map, (e) K_1_^trans^ map, (f) K_2_^trans^ map, (g) k_ep_ map (h) k^1^_ep_ map (i) k^2^_ep_ map.

**Figure 3:**
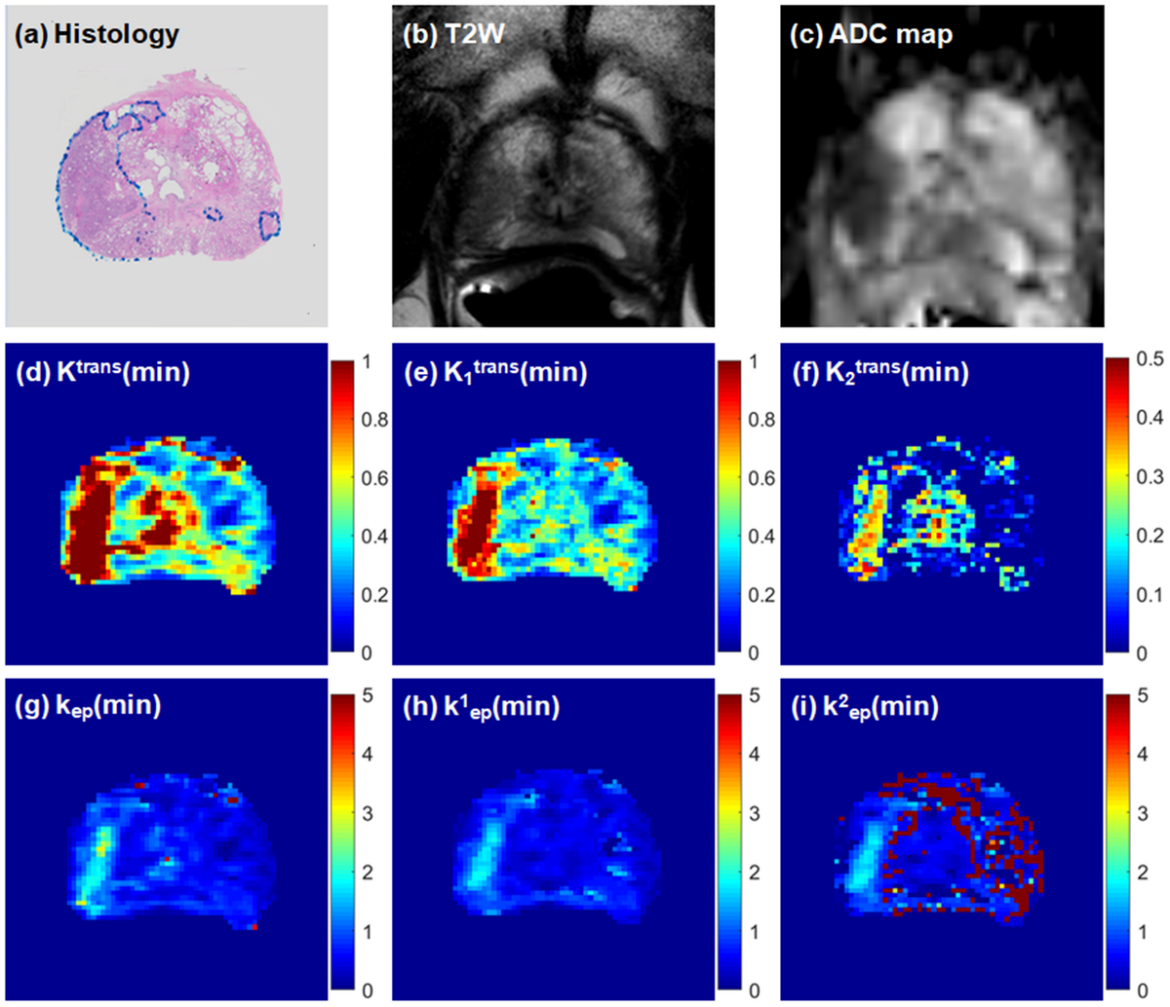
Comparison of parametric maps obtained from the standard Tofts model and 2TCM in a 66-year-old patient with large Gleason 3+4 index lesion: (a) whole-mount histology from prostatectomy specimen with cancer markers, (b) high resolution T2W image, (c) ADC map, (d) K^trans^ map, (e) K_1_^trans^ map, (f) K_2_^trans^ map, (g) k_ep_ map (h) k^1^_ep_ map (i) k^2^_ep_ map.

Figures 2 and 3 show two examples of comparing (a) a histology slice, (b) the corresponding T2W image and (c) ADC map with physiological parametric maps obtained from the standard Tofts model ((d) K^trans^ and (g) k_ep_) and the 2TCM ((e, f) K_i_^trans^ and (h, i) k^i^_ep_, i = 1, 2). In Fig. 2, K_1_^trans^ is similar to K^trans^, and K_2_^trans^ is more specific for cancer. Similarly for Fig. 3, K_1_^trans^ is similar to K^trans^ but with only higher value in cancer region. By comparing with histopathology slice, the K^trans^ and K_1_^trans^ maps show extended cancer better than T2W image and ADC map.

**Figure 4:**
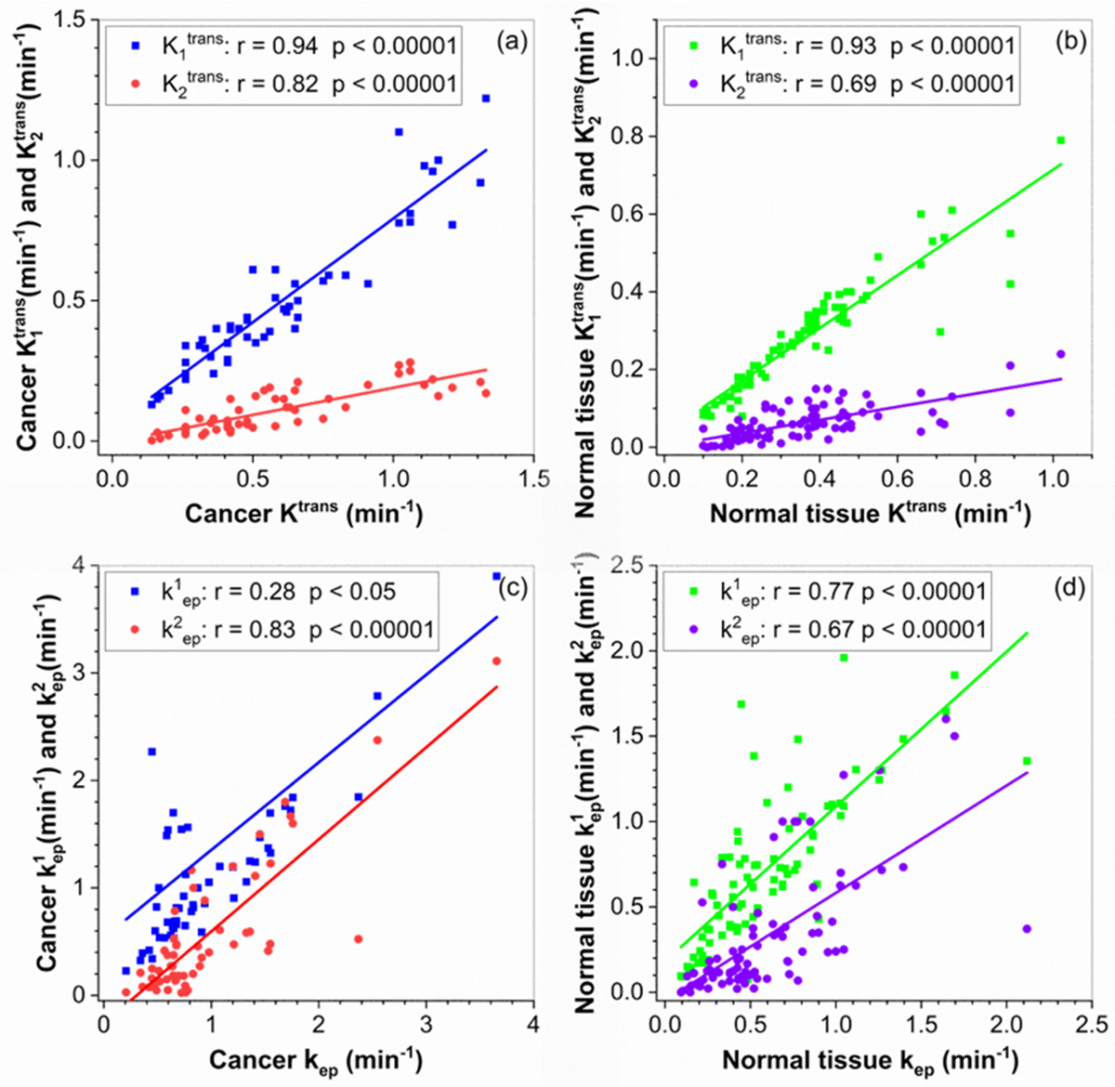
Scatter plots of physiological parameters (K^trans^ and k_ep_) obtained from Tofts model vs. parameters (K_i_^trans^ and k^i^_ep_, i=1, 2) calculated from 2TCM for all ROIs of cancer and normal prostate tissue. The colored lines show linear correlations.

Figure 4 shows scatter plots of averaged physiological parameters over the ROIs obtained from Tofts model and 2TCM for (a, d) cancer and (b, c) normal tissue with K^trans^ and K_i_^trans^ (i = 1, 2) in the top row, and k_ep_ and k’_ep_ (i = 1, 2) in the bottom row. There are strong correlations (r = 0.82 to 0.94, p < 0.0001) between K^trans^ and K_i_^trans^ (i = 1, 2) for cancer (Fig. 4 (a)), and moderate to strong correlations (r = 0.69 to 0.93, p < 0.0001) for normal tissue (Fig. 4 (b)). There was weak correlation (r = 0.28, p < 0.05) between k_ep_ and k^1^_ep_, but strong correlation (r = 0.83, p < 0.0001) between k_ep_ and k^2^_ep_ for cancer (Fig. 4 (c)). There were moderate correlations (0.67 < r < 0.77 p < 0.0001) between k_ep_ and k^i^_ep_ (i = 1, 2) for normal tissue (Fig. 4(d)). This indicates more useful parameters could be obtained and suggesting advantages for the 2TCM.

**Figure 5:**
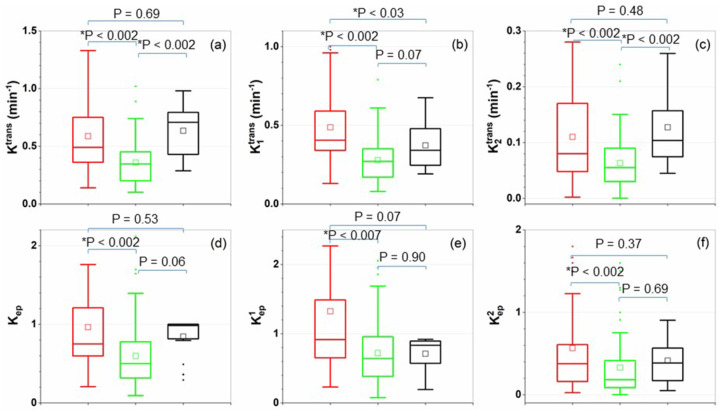
Box-plots of the parametric values obtained from standard Tofts model and 2TCM between ROIs of cancer (red), normal prostate tissue (green) and false positive (black): (a) K^trans^, (b) K_1_^trans^, (c) K_2_^trans^, (d) k_ep_, (e) k^1^_ep_, (f) k^2^_ep_. The square (□) indicates mean. The labeled p-values are calculated from ANOVA with post-hoc Tukey HSD test.

Figure 5 shows box-plots of all physiological parameters compared between cancer (red), normal tissue (green) and selected false positive (black) ROIs for (a) K^trans^, (b) K_1_^trans^, (c) K_2_^trans^, (d) k_ep_, (e) k^1^_ep_, (f) k^2^_ep_. ANOVA with post-hoc Tukey HSD test showed significant difference (p < 0.007) for all the physiological parameters between cancer and normal tissue. There is a clear difference (p < 0.03) between cancer and false positive ROIs for K_1_^trans^, but not for other parameters. When comparing normal tissue with the false positive ROIs, there is significant difference (< 0.002) between K^trans^ and K_2_^trans^, but not for K_1_^trans^. There is no statistical difference (p ≥ 0.06) between normal tissue and false positive ROIs for k_ep_ and k^i^_ep_ (i = 1, 2). By performing receiver operating characteristics (ROC) analysis between cancer and false positive ROIs for the parameters K^trans^ and K_1_^trans^, the area under the curve (AUC) of ROC was 0.43 and 0.63 for K^trans^ and K_1_^trans^, respectively. This indicates that there was no difference between cancer and false positive for K^trans^, but there was a difference for K_1_^trans^.

Finally, Table 1 shows ROC analysis results for all individual parameters and combined parameters obtained from the Tofts model and 2TCM when differentiating between cancer and normal tissue. It shows that the parameter K_1_^trans^ has the highest AUC of 0.787, which is at least ~ 7% higher than any other individual parameter. The combined four parameters obtained from the 2TCM have a much higher AUC value of 0.800 than the combined two parameters obtained from the Tofts model (0.742).

## Discussion

The 2TCM was compared with the Tofts model by using prostate DCE-MRI data. For all calculated parameters, PCa had significantly higher values than normal tissue. Our results also demonstrated that prostate cancer is heterogeneous, involving both the fast (K_1_^trans^) and the slow (K_2_^trans^) exchange compartments. Mathematically, when k^1^_ep_ and k^2^_ep_ are the same, two additive exponential terms can be combined as one exponential term, which is used in the Tofts model. However, Fig. 4 (c) and (d) clearly showed that k^1^_ep_ and k^2^_ep_ are very different. Therefore, it is necessary to have the 2TCM to fit PCa contrast agent concentration curve accurately. The strong correlation between K^trans^ and K_1_^trans^ but weak correlation between k_ep_ and k^1^_ep_ in cancer tissue suggests that the 2TCM is needed in diagnostic PCa. The fast K_1_^trans^ obtained from the 2TCM showed better separation between cancer and normal tissue than K^trans^ obtained from the Tofts model. Combined 2TCM parameters had much a higher AUC value than combined Tofts model parameters, suggesting potential advantages for diagnosis of PCa.

PIRADS version 2.1 favors qualitative analysis of DCE-MRI data, which is the least standardized compared to T2W imaging and DWI [10]. On the other hand, the two-compartment (i.e. blood plasma and EES) pharmacokinetic Tofts model is the most commonly used quantitative analysis technique in clinic for diagnosis of PCa. However, the standard Tofts model often could not fit cancer contrast agent concentration curve accurately and there were false positives when using calculated parameters to detect cancer. Our results demonstrate that the 2TCM could further improve accuracy in diagnosing PCa compared with the Tofts model. This improvement in quantitative analysis of DCE-MRI data would enhance the role of DCE-MRI in prostate mpMRI.

Our results showed that PCa had much higher K^trans^ and k_ep_ than normal prostate tissue, which was consistent with previous studies [24,14]. Similarly, all the parameters calculated from the 2TCM were also much higher in cancer than normal tissue. The fast K_1_^trans^ is similar to K^trans^ in the diagnosis of cancer, but K_1_^trans^ has much lower values at selected false positive regions where K^trans^ is higher.

As we demonstrated in this study, using the 2TCM is just as simple as using the Tofts model. But the results obtained from the 2TCM were much richer than the Tofts model. There were several limitations to this study. First, our sample size was relatively small. The 2TCM should be tested in a much larger and more diverse group of patients. Second, there was no reliable analysis for the drawn ROIs. Since both Tofts model and 2TCM shared the same ROIs, the result of comparisons between two models should be valid. Third, the 2TCM was only compared with the Tofts model. In the future, more models should be compared with 2TCM. Finally, we did not follow the contrast agent for a longer period of time (≤ 8 minutes). If DCE-MRI data were acquired for a longer period, we believe the advantage of the 2TCM would be more apparent obvious, as the errors in fitting curves using the Tofts model would be higher. Nevertheless, more studies are needed to further explore the 2TCM in analysis of prostate DCE-MRI data.

## Conclusion

Our study demonstrated that the 2TCM of DCE-MRI may be useful for quantitative analysis of prostate DCE-MRI. We compared the 2TCM with the standard Tofts model to demonstrate an advantage using the 2TCM. The Tofts model often does not fit contrast agent concentration curves accurately and the 2TCM may provide new diagnostic information in prostate cancer.

## Figures and Tables

**Table 1:** Area under curve (AUC) of receiver operating characteristics (ROC) analysis used to evaluate the performance in differentiating cancer from normal prostatic tissue. Binary logistic regression was used for combined parameters. The 95% confidence interval is shown in brackets.

Model	Parameter	Individual parameter AUC	Combine two parameters AUC	Combine four parameters AUC
**Tofts**	**K** ^**trans**^	0.733 (0.646, 0.819) P < 0.001	0.742 (0.658, 0.826) P < 0.001	NA
	**k**_**ep**_	0.720 (0.635, 0.804) P < 0.001		
**2TCM**	**K**_**1**_^**trans**^	0.787 (0.709, 0.865) P < 0.001	0.790 (0.711, 0.868) P < 0.001	0.800 (0.725, 0.876) P < 0.001
	**k** ^**1**^_**ep**_	0.694 (0.606, 0.783) P < 0.001		
	**K**_**2**_^**trans**^	0.678 (0.583, 0.772) P < 0.001	0.681 (0.587, 0.775) P < 0.001	
	**k** ^**2**^_**ep**_	0.636 (0.540, 0.731) P = 0.007		

## References

[1] Gaunay G, Patel V, Shah P Moreira D, Hall SJ, Vira MA, Schwartz M, Kreshover J, Ben-Levi E, Villani R, Rastinehad A, Richstone L (2017) Role of multi-parametric MRI of the prostate for screening and staging: Experience with over 1500 cases. Asian J Urol 4 (1):68–74. doi:10.1016/j.ajur.2016.09.011 29264209 PMC5730898

[2] Stabile A, Giganti F, Rosenkrantz AB, Taneja SS, Villeirs G, Gill IS, Allen C, Emberton M, Moore CM, Kasivisvanathan V (2020) Multiparametric MRI for prostate cancer diagnosis: current status and future directions. Nat Rev Urol 17 (1):41–61. doi:10.1038/s41585-019-0212-4 31316185

[3] Chatterjee A, He D, Fan X, Antic T, Jiang Y, Eggener S, Karczmar GS, Oto A (2019) Diagnosis of Prostate Cancer by Use of MRI-Derived Quantitative Risk Maps: A Feasibility Study. AJR Am J Roentgenol 213 (2):W66–W75. doi:10.2214/ajr.18.20702 31039019

[4] Mason J, Adiotomre E, Bownes P Carey B, Henry A (2018) Importance of dynamic contrast enhanced magnetic resonance imaging for targeting biopsy and salvage treatments after prostate cancer recurrence. J Contemp Brachytherapy 10 (6):570–572. doi:10.5114/jcb.2018.79667 30662481 PMC6335551

[5] Muller BG, van den Bos W, Brausi M, Fütterer JJ, Ghai S, Pinto PA, Popeneciu IV, de Reijke TM, Robertson C, de la Rosette JJ, Scionti S, Turkbey B, Wijkstra H, Ukimura O, Polascik TJ (2015) Follow-up modalities in focal therapy for prostate cancer: results from a Delphi consensus project. World J Urol 33 (10):1503–1509. doi:10.1007/s00345-014-1475-2 25559111 PMC7721864

[6] Verma S, Turkbey B, Muradyan N, Rajesh A, Cornud F, Haider MA, Choyke PL, Harisinghani M (2012) Overview of dynamic contrast-enhanced MRI in prostate cancer diagnosis and management. AJR Am J Roentgenol 198 (6):1277–1288. doi:10.2214/ajr.12.8510 22623539 PMC6309691

[7] Wu LM, Xu JR, Gu HY, Hua J, Zhu J, Chen J, Zhang W, Hu J (2013) Role of magnetic resonance imaging in the detection of local prostate cancer recurrence after external beam radiotherapy and radical prostatectomy. Clin Oncol (R Coll Radiol) 25 (4):252–264. doi:10.1016/j.clon.2012.11.010 23313568

[8] Wu X, Reinikainen P Kapanen M, Vierikko T, Ryymin P Kellokumpu-Lehtinen PL (2018) Dynamic Contrast-Enhanced Imaging as a Prognostic Tool in Early Diagnosis of Prostate Cancer: Correlation with PSA and Clinical Stage. Contrast Media Mol Imaging 2018:3181258. doi:10.1155/2018/3181258 30327584 PMC6169212

[9] Chatterjee A, He D, Fan X, Wang S, Szasz T, Yousuf A, Pineda F, Antic T, Mathew M, Karczmar GS, Oto A (2018) Performance of Ultrafast DCE-MRI for Diagnosis of Prostate Cancer. Acad Radiol 25 (3):349–358. doi:10.1016/j.acra.2017.10.004 29167070 PMC6535050

[10] Turkbey B, Rosenkrantz AB, Haider MA, Padhani AR, Villeirs G, Macura KJ, Tempany CM, Choyke PL, Cornud F, Margolis DJ, Thoeny HC, Verma S, Barentsz J, Weinreb JC (2019) Prostate Imaging Reporting and Data System Version 2.1: 2019 Update of Prostate Imaging Reporting and Data System Version 2. Eur Urol 76 (3):340–351. doi:10.1016/j.eururo.2019.02.033 30898406

[11] Lovegrove CE, Matanhelia M, Randeva J, Eldred-Evans D, Tam H, Miah S, Winkler M, Ahmed HU, Shah TT (2018) Prostate imaging features that indicate benign or malignant pathology on biopsy. Transl Androl Urol 7 (Suppl 4):S420–S435. doi:10.21037/tau.2018.07.06 30363462 PMC6178322

[12] Ziayee F, Ullrich T, Blondin D, Irmer H, Arsov C, Antoch G, Quentin M, Schimmöller L (2021) Impact of qualitative, semi-quantitative, and quantitative analyses of dynamic contrast-enhanced magnet resonance imaging on prostate cancer detection. PLoS One 16 (4):e0249532. doi:10.1371/journal.pone.0249532 33819295 PMC8021163

[13] Tofts PS, Brix G, Buckley DL, Evelhoch JL, Henderson E, Knopp MV, Larsson HB, Lee TY, Mayr NA, Parker GJ, Port RE, Taylor J, Weisskoff RM (1999) Estimating kinetic parameters from dynamic contrast-enhanced T(1)-weighted MRI of a diffusable tracer: standardized quantities and symbols. J Magn Reson Imaging 10 (3):223–232. doi:10.1002/(sici)1522-2586(199909)10:3<223∷aid-jmri2>3.0.co;2-s 10508281

[14] Cristel G, Esposito A, Damascelli A, Briganti A, Ambrosi A, Brembilla G, Brunetti L, Antunes S, Freschi M, Montorsi F, Del Maschio A, De Cobelli F (2019) Can DCE-MRI reduce the number of PI-RADS v.2 false positive findings? Role of quantitative pharmacokinetic parameters in prostate lesions characterization. Eur J Radiol 118:51–57. doi:10.1016/j.ejrad.2019.07.002 31439258

[15] Franiel T, Ludemann L, Rudolph B, Rehbein H, Staack A, Taupitz M, Prochnow D, Beyersdorff D (2008) Evaluation of normal prostate tissue, chronic prostatitis, and prostate cancer by quantitative perfusion analysis using a dynamic contrast-enhanced inversion-prepared dual-contrast gradient echo sequence. Invest Radiol 43 (7):481–487. doi:10.1097/RLI.0b013e31816b2f63 18580330

[16] Schimpf O, Hindel S, Ludemann L (2017) Assessment of micronecrotic tumor tissue using dynamic contrast-enhanced magnetic resonance imaging. Phys Med 34:38–47. doi:10.1016/j.ejmp.2017.01.010 28139354 PMC5320396

[17] Port RE, Knopp MV, Hoffmann U, Milker-Zabel S, Brix G (1999) Multicompartment analysis of gadolinium chelate kinetics: blood-tissue exchange in mammary tumors as monitored by dynamic MR imaging. J Magn Reson Imaging 10 (3):233–241. doi:10.1002/(sici)1522-2586(199909)10:3<233::aid-jmri3>3.0.co;2-m 10508282

[18] Li X, Priest RA, Woodward WJ, Tagge IJ, Siddiqui F, Huang W, Rooney WD, Beer TM, Garzotto MG, Springer CS Jr. (2013) Feasibility of shutter-speed DCE-MRI for improved prostate cancer detection. Magn Reson Med 69 (1):171–178. doi:10.1002/mrm.24211 22457233 PMC3532861

[19] Brix G, Zwick S, Kiessling F, Griebel J (2009) Pharmacokinetic analysis of tissue microcirculation using nested models: multimodel inference and parameter identifiability. Med Phys 36 (7):2923–2933. doi:10.1118/1.3147145 19673191 PMC2832037

[20] Sourbron SP Buckley DL (2011) On the scope and interpretation of the Tofts models for DCE-MRI. Magn Reson Med 66 (3):735–745. doi:10.1002/mrm.22861 21384424

[21] Sommer JC, Schmid VJ (2014) Spatial two-tissue compartment model fordynamic contrast-enhanced magnetic resonance imaging. Journal of the Royal Statistical Society Series C (Applied Statistics) 63:695–713

[22] Dale BM, Jesberger JA, Lewin JS, Hillenbrand CM, Duerk JL (2003) Determining and optimizing the precision of quantitative measurements of perfusion from dynamic contrast enhanced MRI. J Magn Reson Imaging 18 (5):575–584. doi:10.1002/jmri.10399 14579401

[23] Johnson LM, Turkbey B, Figg WD, Choyke PL (2014) Multiparametric MRI in prostate cancer management. Nat Rev Clin Oncol 11 (6):346–353. doi:10.1038/nrclinonc.2014.69 24840072 PMC6330110

[24] Berman RM, Brown AM, Chang SD, Sankineni S, Kadakia M, Wood BJ, Pinto PA, Choyke PL, Turkbey B (2016) DCE MRI of prostate cancer. Abdom Radiol (NY) 41 (5):844–853. doi:10.1007/s00261-015-0589-3 27193787 PMC6462146

